# Academic Life in Emergency Medicine Blog and Podcast Watch: Toxicologic Emergencies

**DOI:** 10.7759/cureus.3687

**Published:** 2018-12-05

**Authors:** Andrew Grock, Natasha Wheaton, Lynn Roppolo, Chris Gaafary

**Affiliations:** 1 Emergency Medicine, University of California, Los Angeles, USA; 2 Emergency Medicine, University of Texas Southwestern, Dallas, USA; 3 Emergency Medicine, University of South Carolina College of Medicine, Greenville, USA

**Keywords:** aliem, air, toxicology, foam, online education

## Abstract

The Academic Life in Emergency Medicine (ALiEM) Approved Instructional Resources (AIR) Series was created in 2014 to address a lack of both curation of online educational content and a nationally available curriculum that meets individualized interactive instruction. Using an expert-based, crowdsourced approach, the AIR series identifies trustworthy, high-quality, educational blog and podcast content.

Here, we summarize the content rated as high quality per our a priori criteria as evaluated by eight attending physicians.

## Introduction and background

Emergency medicine (EM)-directed online educational content has risen dramatically, though quality assessment of these resources is far less robust [1-4]. Additionally, the 2008 Accreditation Council for Graduate Medical Education recommendations included the option to decrease synchronous conference by up to 20% in exchange for asynchronous learning termed individualized interactive instruction (III) [5].

To address these needs, the Academic Life in Emergency Medicine (ALiEM) Approved Instructional Resources (AIR) Series was created in 2014 to help EM residency programs identify quality online content on social media [6-7]. For the purpose of formally presenting our curated quality resources, the ALiEM series editorial board summarizes each topic in the Blog and Podcast Watch series [8-9]. This installment summarizes the highest scoring online educational resources on toxicology emergencies.

## Review

Topic identification

The AIR Series is a continuously building curriculum.

Inclusion and Exclusion Criteria

Relevant content from the previous 12 months was collated from the 50 most impactful sites per the Social Media Index [10]. The search, conducted in January 2017, included blog posts and podcasts written in English for scoring by our expert panel.

Scoring

Eight reviewers from the AIR Editorial Board, EM core faculty from various United States medical institutions scored each post without blinding. The scoring rubric contains five measurements, each divided into a seven point scale: Best Evidence in Emergency Medicine (BEEM) score, accuracy, educational utility, evidence-based, and references (Table 1) [11]. More detailed methods are described in the original description of the AIR Series [6-7]. Board members with any role in producing a resource recused him/herself from evaluating that resource.

**Table 1:** Approved Instructional Resources scoring instrument for blog and podcast content. BEEM: Best Evidence in Emergency Medicine EP: Emergency Physician

Tier 1: BEEM Rater Scale	Score	Tier 2: Content accuracy	Score	Tier 3: Educational Utility	Score	Tier 4: EBM	Score	Tier 5: Referenced	Score
Assuming that the results of this article are valid, how much does this article impact on EM clinical practice?		Do you have any concerns about the accuracy of the data presented or conclusions of this article?		Are there useful educational pearls in this article for residents?		Does this article reflect evidence-based medicine (EBM)?		Are the authors and literature clearly cited?	
Useless information	1	Yes, many concerns due to many inaccuracies found	1	Not required knowledge for a competent EP	1	Not EBM based, only expert opinion	1	No	1
Not really interesting, not really new, changes nothing	2		2		2		2		2
Interesting and new, but doesn't change practice	3	Yes, a major concern about a few inaccuracies	3	Yes, but there are only a few (1-2) educational pearls that will make the EP a better practitioner to know or multiple (>=3) educational pearls that are interesting or potentially useful, but rarely required or helpful for the daily practice of an EP.	3	Minimally EBM based	3		3
Interesting and new, has the potential to change practice	4		4		4		4	Yes, authors and general references are listed (but no in-line references)	4
New and important: this would probably change practice for some EPs	5	Minimal concerns, only minor inaccuracies found	5	Yes, there are several (>=3) educational pearls that will make the EP a better practitioner to know, or a few (1-2) every competent EP must know in their practice	5	Mostly EBM based	5		5
New and important: this would change practice for most EPs	6		6		6		6		6
This is a "must know" for EPs	7	No concerns, no inaccuracies found	7	Yes, there are multiple educational pearls that every competent EP must know in their practice	7	Yes exclusively EBM based	7	Yes, authors and in-line references are provided	7
Your Score									

Resources with a mean evaluator score greater than 30 points (out of a maximum of 35) are awarded AIR accreditation. Resources with a mean score of 27 to 29 are given the Honorable Mention (HM) label.

Results

We initially included a total of 145 blog posts and podcasts. Key educational pearls from the six Honorable Mentions are described. There were zero AIR posts for this topic.

Article 1: Sisson M and Culver M. Bark Scorpion Sting: Indications for Anascorp and dosing controversies. Academic Life in Emergency Medicine. (July 27, 2016) HM

https://www.aliem.com/2016/07/bark-scorpion-bite-anascorp/

This blog post reviews envenomation by the Bark Scorpion (genus Centruroides).

Take-Home Points

Bark Scorpion envenomation results in sympathetic and parasympathetic storm that can present with involuntary jerking, cranial neuropathies (e.g., wandering eye movements), myocardial damage, and most threatening – loss of airway. While mild envenomations present with pain and paresthesias at or remote from the bite site, severe envenomations present with myocardial damage, loss of airway, neuromuscular symptoms such as jerking movements, or cranial nerve dysfunction such as slurred speech or difficulty talking. Children and the elderly are at greatest risk for severe complications. Treatment is mainly supportive and includes benzodiazepines, opioids and intubation for any upper airway dysfunction or difficulty with secretions. Severe envenomations should receive antivenin, called Anascorp, though dosing is complicated. The federal drug administration (FDA) recommends administering three vials initially, but, if the patient does not require immediate intubation, alternate dosing is frequently recommended. Administer one vial followed by 30 to 60 minutes prior to re-dosing. Keep in mind, each vial costs at least three thousand dollars in the United States [12].

Article 2: Swaminathan A. Baclofen Withdrawal. REBEL EM. (Oct 27 2016) HM

http://rebelem.com/baclofen-withdrawal/

This blog post reviews the often overlooked but potentially life-threatening pathology of baclofen withdrawal.

Take-Home Points

Baclofen, a gamma-aminobutyric acid (GABA) receptor agonist, decreases muscle tone and spasticity. Signs and symptoms of withdrawal typically occur 24 to 48 hours after the last baclofen dose and includes the following symptoms: increased spasticity, fever, and neuropsychiatric symptoms like confusion and altered mental status. Withdrawal is also associated with hypertension, hyperthermia, tachycardia, seizures and rhabdomyolysis. Baclofen pumps, which administer it intrathecally, may result in acute withdrawal if the pump dislodges, is underfilled, or malfunctions. Pump mechanical dysfunction may be visualized with an abdominal X-ray. Also, neurosurgery, interventional pain management, or rehabilitation medicine can interrogate the pump. Withdrawal management includes: fluid resuscitation, benzodiazepines for seizure control, aggressive cooling if needed, screening labs (including a creatinine kinase (CK) to evaluate for rhabdomyolysis), and, more obviously, the administration of baclofen. For intrathecal pump-induced withdrawal, oral baclofen is unlikely to be helpful. Intrathecal baclofen can be given via the pump or lumbar puncture, though these interventions are generally performed by neurosurgery. Propofol, along with an advanced airway, may also temporize the patient until definitive therapy is available. Baclofen withdrawal is life-threatening and most patients suffering from this condition should be admitted to the intensive care unit (ICU) [13].

Article 3: Hughes D. Benzodiazepine Refractory Alcohol Withdrawal. REBEL EM. (April 28, 2016) HM

http://rebelem.com/benzodiazepine-refractory-alcohol-withdrawal/

This post provides an evidence-based review of adjunct treatments for benzodiazepine-refractory withdrawal.

Take-Home Points

In addition to supportive care, benzodiazepines are the mainstay of alcohol withdrawal treatment. Surprisingly, some withdrawals are refractory to benzodiazepines as defined by greater than 10 mg lorazepam equivalents administered within one hour or 40 lorazepam equivalents in four hours. Of the adjuvant therapies for benzodiazepine-refractory withdrawal (phenobarbital, propofol, ketamine and dexmedetomidine), all but ketamine have been shown to reduce benzodiazepine requirements. However, no treatment exists that can shorten the duration of alcohol withdrawal [14].

Article 4: Helman A, Thompson M, Austin E. Low and Slow Poisoning. Emergency Medicine Cases. (January 2017) HM

https://emergencymedicinecases.com/low-slow-poisoning/

This post by Emergency Medicine Cases excellently reviews the toxicologic patients with hypotension and bradycardia.

Take-Home Points

For patients with bradycardia and hypotension consider both non-toxicologic (myocardial infarction (MI) with cardiogenic shock, hyperkalemia, myxedema coma, spinal cord injury and hypothermia) and toxicological (calcium channel blockers (CCB), beta-blockers (BB), digoxin, opiates, alpha-2 antagonists, sodium channel blockers, cyclobenzaprine, and antipsychotics) etiologies. Mentation and glucose can differentiate CCB from BB toxicity. Typically BB overdoses present with obtundation and normal to low glucose while CCB overdoses have a normal mental status with hyperglycemia. For seizures, benzodiazepines are superior to other antiepileptic drugs which may have potentially dangerous sodium channel blockade effect. For sodium channel blocker toxicity, which may present with a terminal R in AVR or a wide QRS, administer sodium bicarbonate. If less than one hour from time of ingestion, charcoal may decrease drug bioavailability. This restriction can be extended in specific circumstances – able to protect the airway, low seizure risk, and extended release drug or delayed drug absorption. Gastric lavage should be considered only for massive life-threatening ingestions, if it can be performed within one hour of ingestion. Glucagon should be given as a last resort as it can cause vomiting and worsen the patient’s condition. For digoxin toxicity, digoxin immune FAB (digifab) is recommended if: hyperkalemia, > 10 mg ingested in an adult or > 4 mg in a child, renal failure, unstable dysrhythmias, acute ingestion with serum digoxin level >12 nanogram/mL, or multiple drug ingestions [15]. Transvenous pacing may precipitate dysrhythmias in digoxin overdose. For the “low and slow” patient, intravenous fluid boluses, atropine, calcium, high dose regular insulin, lipid emulsion therapy and vasopressors (epinephrine preferred if poor cardiac contractility) can be helpful. Extracorporeal membrane oxygenation (ECMO) should be considered if the patient is refractory to other aggressive treatments [16].

Article 5: Misch M. CritCases 1: Massive TCA Overdose. Emergency Medicine Cases. (January 2016)

https://emergencymedicinecases.com/critcases-massive-tca-overdose/

This blog reviews the management of massive tricyclic antidepressant toxicity (TCAt).

Take-Home Points

Though relying on expert consensus given most of the evidence is animal based or case report based, treatment for TCAt includes: sodium bicarbonate, hypertonic saline, intralipid, lidocaine, norepinephrine, activated charcoal, whole bowel irrigation, and extra-corporal membrane oxygenation. TCAt treatment is indicated in patients with QRS > 100 msec, seizures, and cardiac arrest. Sodium bicarbonate boluses with an initial dose of 100-150 mEq intravenous push is first line and up to 4 amps (200 mEq) can be given. After, a sodium bicarbonate drip (100-150 mEQ in 1 L of D5W at 250 ml/hr) can be given. Hourly electrolyte and blood gases can help keep the pH < 7.55 and sodium < 155 mmol/L. Intra-lipids (1.5 ml/kg over one minute followed by 0.25 ml/kg/hr up to 2 ml/kg) are recommended for patients with hemodynamic instability, ventricular arrhythmias or refractory seizures despite adequate sodium bicarbonate administration. This dose can be repeated if needed. Hypertonic saline (100 ml), rather than continued sodium bicarbonate, is recommended if the pH is above 7.55 but the sodium is still less than 150-155 mmol/L. If serum sodium is the limiting factor ( >150-155 mmol/L), lidocaine should be given at 1-1.5 mg/kg up to 150 mg. Either norepinephrine or epinephrine are appropriate pressor choices. If ventilated, hyperventilation can help reach the goal pH of 7.5-7.55. Lastly, if all else fails, ECMO may be helpful for refractory unstable arrhythmias or hemodynamic instability [17].

Article 6: Hayes B. ‘Treat and Release’ after Naloxone - What is the Risk of Death? ALiEM. (August 24, 2016)

https://www.aliem.com/2016/08/treat-and-release-after-naloxone/

This post evaluates an analysis of death rates in patients who refuse transport after receiving naloxone in the prehospital setting.

Take-Home Points

Of the 205 deaths that occurred within six months of a prehospital encounter involving naloxone administration with subsequent refusal of care, only one (0.49%) occurred within 24 hours of the prehospital encounter. Ample comments emphasize that this study was retrospective and only looked at patient deaths. While this data does suggest that a patient is low risk for short term poor outcomes (recurrent respiratory depression and death) after receiving naloxone, ED transport and the standard four- to six-hour observation is still recommended. Additionally, this study may not be generalizable to populations with different mixes of narcotics used [18].

## Conclusions

The ALiEM Blog and Podcast Watch Series serves to identify high-quality educational blogs and podcasts for EM clinicians. The resources curated specifically for toxicology diseases are herein shared and summarized to help clinicians filter the rapidly published multitude of blog posts and podcasts. Our search was limited to content produced within the previous 12 months from the top 50 Social Media Index sites. While these lists are by no means a comprehensive analysis of the entire internet for this topic, this series has identified the above online resources as the highest quality. While this article focuses on toxicology diseases, additional AIR modules address other topics in emergency medicine.
